# Fiber Optic Sensor of Ammonia Gas Using Plasmonic Extraordinary Optical Transmission

**DOI:** 10.3390/s23084065

**Published:** 2023-04-18

**Authors:** Ladislav Kalvoda, Jaroslava Jakoubková, Milan Burda, Pavel Kwiecien, Ivan Richter, Jaromír Kopeček

**Affiliations:** 1 Faculty of Nuclear Sciences and Physical Engineering, Czech Technical University in Prague, Břehová 7, 115 19 Prague, Czech Republic; jaroslava.jakoubkova@fjfi.cvut.cz (J.J.); milan.burda@fjfi.cvut.cz (M.B.); pavel.kwiecien@fjfi.cvut.cz (P.K.); ivan.richter@fjfi.cvut.cz (I.R.); 2 FZU—Institute of Physics of the Czech Academy of Sciences, Na Slovance 1999/2, 182 21 Prague, Czech Republic; kopecek@fzu.cz

**Keywords:** ammonia gas sensor, localized plasmon, nanohole array, extraordinary light transmission, Fourier modal method, organometallic complex reagent, quinoline derivatives

## Abstract

While standard surface plasmon resonance (bio) sensing, relaying on propagating surface plasmon polariton sensitivity on homogeneous metal/dielectric boundaries, represents nowadays a routine sensing technique, other alternatives, such as inverse designs with nanostructured plasmonic periodic hole arrays, have been far less studied, especially in the context of gas sensing applications. Here, we present a specific application of such a plasmonic nanostructured array for ammonia gas sensing, based on a combination of fiber optics, extraordinary optical transmission (EOT) effect, and chemo-optical transducer selectively sensitive to ammonia gas. The nanostructured array of holes is drilled in a thin plasmonic gold layer by means of focused ion beam technique. The structure is covered by chemo-optical transducer layer showing selective spectral sensitivity towards gaseous ammonia. Metallic complex of 5-(4′-dialkylamino-phenylimino)-quinoline-8-one dye soaked in polydimethylsiloxane (PDMS) matrix is used in place of the transducer. Spectral transmission of the resulting structure and its changes under exposition to ammonia gas of various concentrations is then interrogated by fiber optics tools. The observed VIS-NIR EOT spectra are juxtaposed to the predictions performed by the rigorous Fourier modal method (FMM), providing useful theoretical feedback to the experimental data, and ammonia gas sensing mechanism of the whole EOT system and its parameters are discussed.

## 1. Introduction

Gaseous ammonia has a chemical formula NH_3_, a molecular weight 17,031 g/mol, and dipole moment 1.42 D [1]. Thanks to its electro-positivity, an ammonium cation is easily produced, showing alkaline behavior in an aqueous environment.

Gaseous ammonia is one of the gases we can consider as a normal part of our environment. It is one of the metabolites produced by the rotary bacterium, and, for example, in a mixture of gases exhaled by humans in indoor conditions, it is contained at concentrations of 10–70 ppb [2]. It is worth noting that, in the latter case, the ammonia content provides important information on metabolic processes in the body and is an indicator of a number of severe diseases such as lung cancer.

At an industrial scale, ammonia gas is synthesized by catalytic Haber–Bosch reaction from N_2_ and H_2_ and further used as a starting reactant for the production of nitrates and ammonium salts. Thanks to its gas/liquid phase transformation critical points at temperature 405.4 K and pressure 11.3 MPa [3], ammonia gas is used as a cooling medium in large-scale freezing systems (food storage, cooling trucks and boats, artificial skating rinks and hockey halls, etc.). Significant release of ammonia gas also occurs in farming (poultry or cattle breeding).

The human sense of smell perceives (unpleasantly) the presence of ammonia at concentrations of ca. 50 ppm in the air. At higher concentrations, ammonia gas acts as irritating to the breathing apparatus. Concentration levels of ca. 5000 ppm and higher are considered dangerous to fatal. Ammonia poisoning is manifested by a nerve paralysis of muscles, preventing the affected victim from leaving the place of exposure [4].

Quantitatively exact, sensitive, reversible, and chemically selective detection of gaseous ammonia is thus needed for many practical reasons, among which belong primarily systems of early warning of ammonia leaks in industrial production localities, and also precise and continuous analysis of the content of ammonia in breath implemented as one of the possible functions of a personal physical function monitor. Major detection principles used recently in ammonia gas sensing (involving electric conductometry, electrochemistry, tunable diode laser spectroscopy, surface acoustic waves analysis, field effect transistor structures, and carbon nanomaterials—cf. e.g., the recent reviews [5,6,7]) comply with the requirements of industrial applications; for a wider employment in personnel medicine, however, the current detectors are still unsatisfactory, both in the concentration sensitivity and temporal response speed, and also in the sensing selectivity and reversibility.

One of the ways in which to increase the detector sensitivity limit is to use the high level of electric field gain provided by the excitation of electronic plasmon oscillations in metals (further on, we restrict exclusively to optical excitation of plasmon oscillations). By placing a suitably responding transducer into a space with a high level of the plasmonic evanescent field, a significant reinforcement of the transmitter response can be achieved based on the continuity of the electric field back to the optical excitation and registration system. This physical principle has been developed in a number of sophisticated detection schemes in the last 50 years and new properties are still being discovered [8,9,10,11,12]. The plasmonic detection schemes that can be, in principle, divided into two groups are: the systems relying on surface plasmon polaritons excited on the continuous metal surface (propagating surface plasmons), and the systems in which local plasmonic modes occur in isolated nanostructures (localized surface plasmons). The design of the sensing element presented in this contribution is based on the use of the second of these two options. Specific target gas sensitivity is ensured by the use of organometallic reagent successfully applied in our previous studies dealing with ammonia gas detection by means of sensors based on modified optical fibers [13,14].

## 2. Materials and Methods

### 2.1. Preparation and Characterization of Sensing Structures

Deposition system HVD RHVm42 (Hoch Vakuum Dresden, Dresden, Germany) was employed in preparation of starting Au plasmonic layers by physical vapor deposition (PVD) method in vacuum (5 × 10^−5^ Pa) from Au melt prepared in a Mo electrically heated boat. The layers with thickness within 50–60 nm were deposited on glass BK-7 substrates covered by an intermediate Cr layer. The raw metals were supplied by Sigma-Aldrich s.r.o., Prague, Czech Republic. The layers’ thickness was checked by the Fresnel fit to the attenuated total reflection (ATR) spectrum containing the minimum corresponding to the resonant surface plasmon polariton (SPP) excitation. The measurements were performed in monochromatic Kretschmann configuration (λ = 633 nm) on our home-made ATR system [14]. Dielectric permittivity value ε = 11.8 + 1.5i was used in a Fresnel fitting procedure for the Au layer.

**Figure 1:**
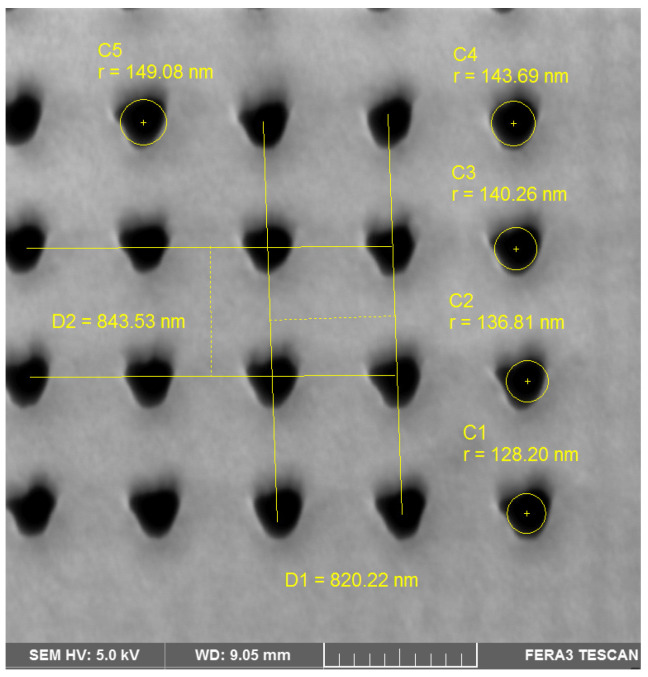
SEM micrograph of a corner part of the grid drilled in Au layer; the main dimensional parameters are shown and measured (SEM voltage 5 kV, SE imaging mode, magnification 50,000×); the shown scale is 1 μm long.

A periodic rectangular grid of nanoholes was then drilled in the prepared Au layers by focused ionic beam (FIB) method. The drilling was performed on the TESCAN Ferra 3 scanning electron microscope (SEM) equipped with Xe-FIB device (Tescan Orsay Holding a.s., Brno, Czech Republic). After necessary optimization of the drilling parameters, the final 200 × 200 square grid of nanoholes was obtained with the diameter and perpendicular translation periods set to 300 nm and 830 nm, respectively. A detailed view of one of the obtained grid corners is shown in Figure 1. Certain imperfections in the drilled structure shape and dimensions are apparent.

Using a standard spin-coating technique, the Au layer with the prepared nano-grid was then covered by a thin layer composed of polydimethylsiloxane (PDMS). Thermally cured PDMS formulation Sylgard 184 (Dow Corning, Midland, MI, USA) was used. The mean thickness 160 ± 10 nm of the PDMS layer was obtained from atomic force microscope (AFM) scan performed over a scratch made in the PDMS slab beside the nano-grid area. LiteScope AFM (NenoVision s.r.o., Brno, Czech Republic) equipped with Akyiama self-sensing probe was used.

The PDMS coating covering the nano-grid was then sensitized by soaking the selected organometallic reagent consisting of Co^2+^ complex with 5-(4′-dioctylamino-phenylimino)-quinoline-8-one ligands (Co-DPQ) in the PDMS buffer, providing the required sensitivity of the whole sensing structure to ammonia gas. More details about the reagent synthesis, PDMS sensitization procedure, optical properties, and reaction mechanism of the reagent with ammonia gas have been given in our former reports [13,14].

### 2.2. Experimental Setup Used to Test Response to Ammonia Gas

**Figure 2:**
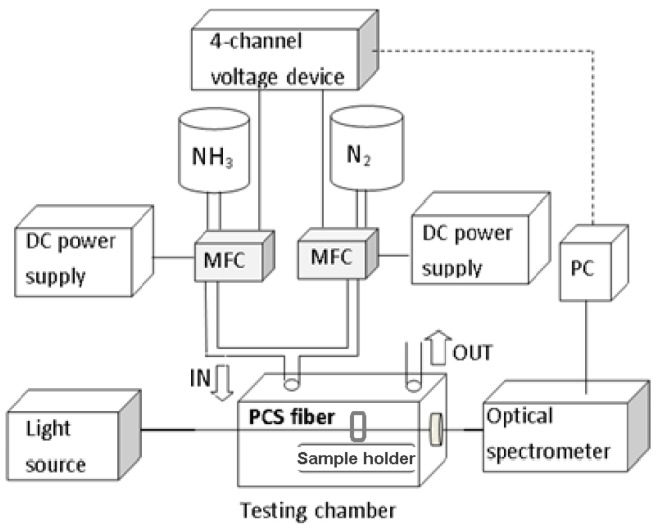
Schema of the experimental setup used in testing the reaction of the sensing structure to ammonia gas; for details see the text.

The schema of experimental setup used to characterize the spectral properties of the prepared structure and its reaction to the presence of ammonia gas of different concentrations in the nitrogen carrier is shown in Figure 2.

The optical setup used involves a white wideband deuterium/wolfram light source (Heraeus FiberLight DTM 6/10 UV/VIS Lamp module; Heraeus Noblelight GmbH, Hanum, Germany), sample holder providing X-Y micrometric positioning, illuminating and collecting fiber optic ports equipped with cylindrical lenses (Newport LGI 830-6, Irvine, CA, USA), and fiber optic spectrometer covering the spectral range 200–1200 nm (S1000, Ocean Optics, Orlando, FL, USA). All the components are optically interconnected by PCS multimode optical fiber (SQS SMA/PCF-S 900 μm patch-cord, Nová Paka, Czech Republic). The optical transmittance spectra measured on the tested grid region are referred to the transmittance of the sample part covered with the continuous gold layer.

The gas preparation and delivering setup used in experiments consists of NH_3_ and N_2_ pure gas sources (pressure cylinders; purity class 4.5; Linde Gas a.s., Prague, Czech Republic), pressure reduction valves, gas mass flow controllers (MFC; UFC 1661C; Celerity Fluid Systems, Dublin, Ireland), a gas exposition glass bell covering the sample holder, and interconnecting stainless tubes. Before recording the observed spectra, each preset gas concentration was released to blow through the exposition chamber for 20 min, in order to achieve a steady state of the tested sample. Operation of both setup parts is fully controlled by a dedicated PC SW.

## 3. Theoretical Part

### 3.1. Basics of Plasmonic Extraordinary Optical Transmission

Extraordinary optical transmittance (EOT) of a plasmonic structure formed by a two-dimensional (2D) periodic array of holes produced in a plasmonic layer of metal (such as Au, Ag) was first described by Ebbesen [15]. This first pioneering work dealing with EOT was later followed by other studies devoted to the topic of enhanced light transmission through one-dimensional (1D) [16] and 2D [17] periodic arrays of slits and holes of subwavelength characteristic size. A number of complementary and, in some cases, conflicting interpretations of the origin of this phenomenon have gradually been proposed. A significant role has been attributed to the resonant interaction of light with the surface plasmon polariton propagating on the metal surface, mediated by the existence of a periodic structure ensuring phase-matching between the incident light and the SPP. This concept is consistent with the shape of the dispersion relation of the SPP, which corresponds to the spectral positions of the EOT [18]. Gradually, also with the contribution of the authors of this paper [19,20], the present established theory of EOT has emerged based on a combination of several processes involved in subwavelength transmission: non-resonant processes, related to the simple passage of light through subwavelength apertures, and resonant ones, taking into account the interaction of the incident light with the periodic arrangement of the apertures. The approaches/phenomena used include (i) the excitation of cavity modes (CM) inside individual holes/slits, the latter considered as open Fabry–Perot (FP) resonators, (ii) the resonant tunneling phenomenon consisting in the coupling of the SPP on both horizontal surfaces of the metallic lattice structure via evanescent waves passing through the holes, (iii) the existence of a “creeping” wave [21], and (iv) the effective medium theory (EMT) approach.

Apart from such microscopic theory, the rigorous treatment of the EOT analysis is, in general, performed using the rigorous numerical technique. In our case, due to the structural periodicity, our in-house software package is effectively used with the implemented Fourier Modal Method (also traditionally called the rigorous coupled-wave analysis, RCWA) [22,23].

### 3.2. Optical Model of the Studied Sensing Structure

In order to interpret the obtained experimental data, plasmonic EOT spectrum is theoretically simulated by the FMM method [23]. The FMM technique is currently considered as one of the most efficient and accurate electromagnetic analysis frameworks for optics and photonics, in terms of allowing general structure modulation, oblique incidence, and analysis of sharp resonance, as required in this paper. We have relied on our previous profound experience with both periodic (1D and 2D cases) and aperiodic (i.e., isolated structures—for 2D and 3D cases) versions of the FMM technique, with several important technical extensions, such as proper Fourier factorization, adaptive spatial resolution [23], the normal vector method, symmetrization techniques, and recently also non-locality [24], applied extensively to various rather complex problems, including, e.g., high-Q optical nanocavities [25], plasmonic gratings, metasurfaces, and waveguides [26], plasmonic sensor structures [27], magneto-optic structures [28], periodic arrays exhibiting the EOT effects [29,30], bound modes in the continuum [29], or graphene plasmons [30,31].

**Figure 3:**
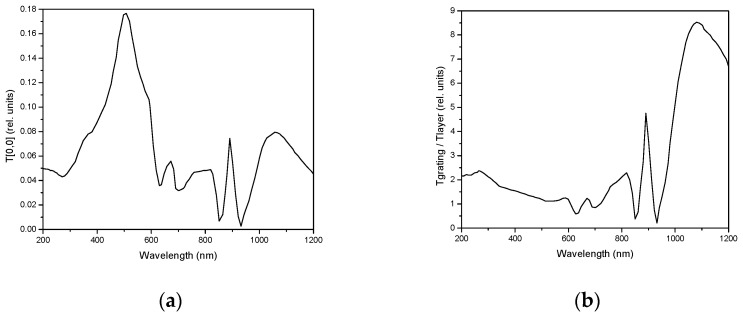
Calculated spectral transmittance of the 2D regular rectangular plasmonic holes array (see text for parameters) referred to BK-7 glass slide (**a**) and continuous Au/Cr/Bk-7 slab (**b**); FMM simulation technique applied.

The following structure is considered for the theoretical analysis and prediction of the properties: a regular rectangular array of 441 cylindrical holes with the diameter 300 nm and the period 830 nm, made in the metallic slab formed by Au layer (51 nm) with the intermediate Cr layer (1.5 nm) prepared on (infinite) BK7 glass substrate, covered by (infinite) PDMS superstrate. Perpendicular incident light beam orientation is considered. The calculated EOT spectrum is shown in Figure 3. As can be seen in Figure 3a, the spectral transmittance T [0, 0] shows a significant resonances in the spectral region of 400–600 nm and also of 1000–1200 nm. When taken relatively, with respect to the response of a continuous Au/Cr/Bk-7 slab (Figure 3b), this second resonance is enhanced, reaching the values of the enhancement over this layer close to 9. In the region of small wavelengths, there is a slight maximum around 300 nm, in good correspondence with the observed experimental data. We have also compared the effect of actual realistic shapes of the subwavelength holes (see Figure 1) with the idealized circular shapes, finding only very small differences located in the spectral region around 700 nm, out of the two resonant areas.

Note that the position of spectral transmittance maxima depends significantly on the regularity, translation period, hole shape, and hole diameter of the metallic structure, as well as on the angle of incidence and polarization state of the primary light beam rays. The theoretical and experimental discussion of these topics is the subject of a number of original publications (see e.g., [15,32,33,34,35,36,37]). Approximately, prolonging the hole lattice period results generally in a red shift of the observed spectral transmission patterns [36]; reducing the hole diameter increases the strengths of EOT modes linked to surface plasmon polaritons, but leads also to their broadening and a slight blue shift [37].

**Figure 4:**
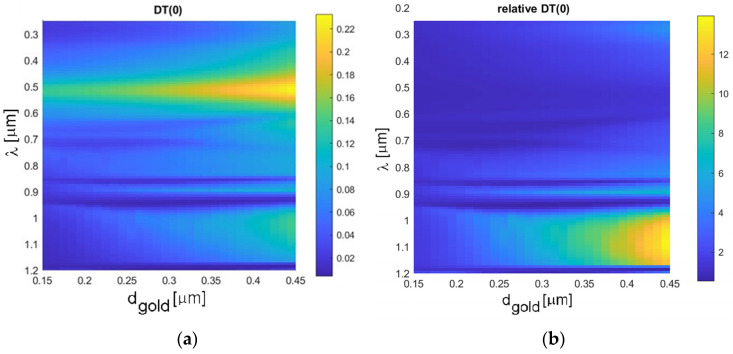
Contour graphs of spectral transmittance calculated by the FMM method for the 2D regular rectangular plasmonic holes array (with the same translation parameters as used in Figure 3), as a function of the gold layer thickness (d_gold_) and wavelength (λ), referred to BK-7 glass slide (**a**) and continuous Au/Cr/Bk-7 slab (**b**).

Figure 4 then shows the calculated 2D contour graphs of spectral transmittance of 2D regular rectangular plasmonic holes arrays (with the same translation parameters as those used in Figure 3), as a function of the gold layer thickness d_gold_ and wavelength. The sensitivity of the spectral transmittance to the actual thickness of the gold layer is apparent. Also, for large thicknesses, the resonant maximum becomes slightly broader. Figure 4b shows the relative transmission enhancement compared to continuous Au layer, with the relative increase of the second (long-wavelength) maximum.

### 3.3. Sensing Mechanism

As we previously confirmed, the used organometallic reagent Co-DPQ provides selective sensitivity to ammonia gas exposition [8], the latter resulting in decomposition of the original reagent complex and freeing the quinoline ligand (*L*) molecules.

(1) $${\left[nL-Co\right]}^{2+}+2Br^{-}+mNH_{3}\Leftrightarrow {\left[{\left(NH_{3}\right)}_{m}-Co\right]}^{2+}+2Br^{-}+nL$$

Here, *m* and *n* denote the corresponding coordination numbers of ammonia and DPQ in the cobalt complex. Related changes of electronic structure lead to a blue shift of the optical absorption band in the visible region. Clearly, the particular spectral positions of the absorption bands corresponding to the initial complex and resulting free ligand depend on the permittivity of the surrounding matrix; in PDMS, the respective centroid/FWHM values are ca. 825/200 nm and 610/150 nm [13]. It is worth noticing that the mentioned simplified reaction schema (1) can be, in reality, more complex, involving, e.g., possible presence of water or chlorine groups appearing at ligand positions.

## 4. Results and Discussion

**Figure 5:**
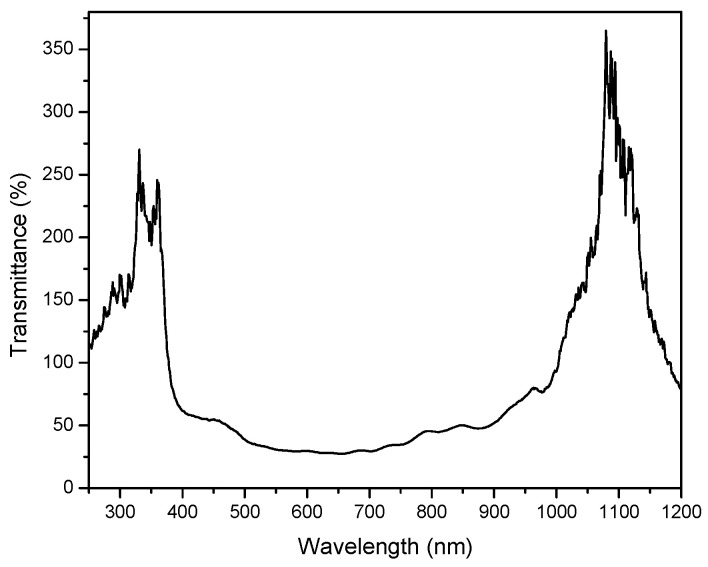
Initial spectral transmittance observed on the tested sensing structure under pure N_2_ atmosphere; plotted values are relative to the continuous Au layer.

The initial transmittance (T) spectrum recorded under pure N_2_ atmosphere on the tested structure is shown in Figure 5. There are two extremes corresponding to extraordinary optical transmission of the Au grid situated at low (ca. 360 nm) and high (ca. 1080 nm) wavelengths. It is worth noticing that both observed maxima positions might be slightly influenced by constituting parts of the used experimental setup, in particular by the absorption edge of the BK-7 substrate and the light emission limit of the wolfram source on the short- and long-wavelength sides of the spectrum, respectively.

**Figure 6:**
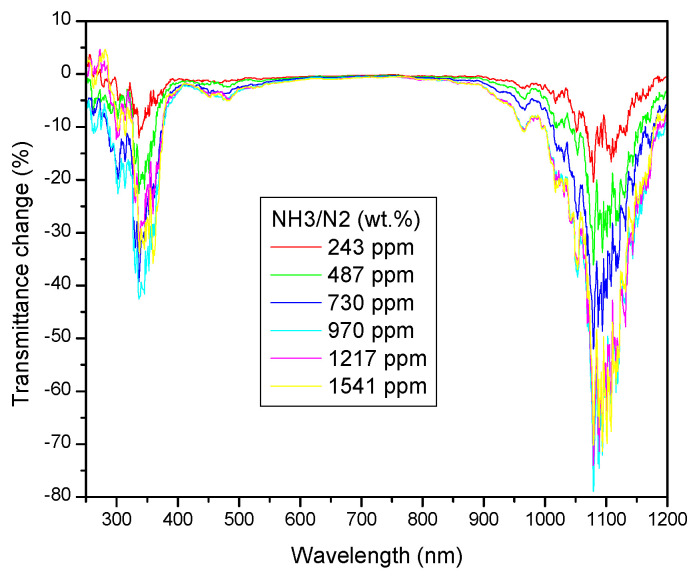
Absolute changes in the spectral transmittance observed under subsequent expositions to NH_3_/N_2_ gas mixtures of the indicated composition.

Figure 6 shows absolute spectral changes in transmittance (ΔT^abs^ = T^NH3/N2^ − T^N2^) under exposition to NH_3_/N_2_ gas mixtures of different mass concentration. Apparently, a decrease in the transmittance is observed at the short- and long-wavelength limits of the scanned spectral interval. The observed transmittance drop is proportional to the ammonia gas concentration increase. At c ≈ 1000 ppm, a saturated state is achieved. The observed spectral regions of the most remarkable absolute transmittance changes clearly overlap with the spectral regions of the maximal transmittance of the tested structure (cf. Figure 5 and Figure 6).

**Figure 7:**
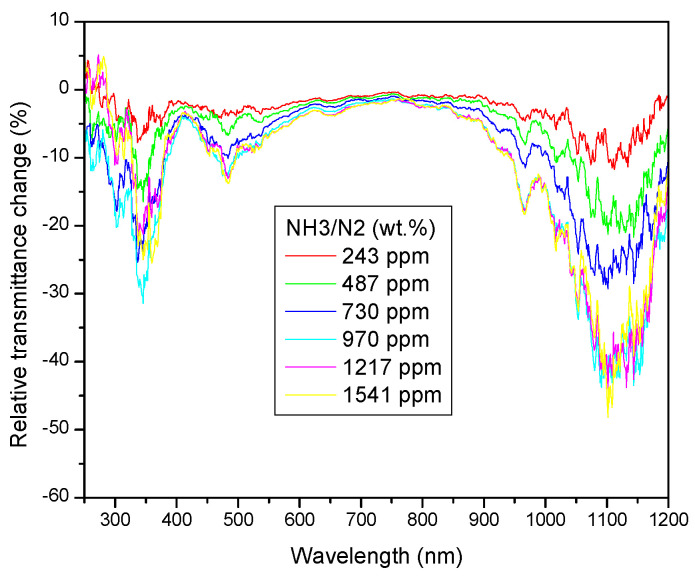
Relative changes in the spectral transmittance observed under subsequent expositions to NH_3_/N_2_ gas mixtures of the indicated composition.

Changing from absolute to the relative transmittance (ΔT^rel^ = ΔT^abs^/T^N2^), the details of spectral changes become even more apparent (Figure 7). The split of the short-wavelength transmittance band is well resolved, with the first stronger extreme of transmittance change at ca. 350 nm, and the second one at ca. 480 nm. At the long-wavelength side, a broad extreme dominates, centered on about 1100 nm, accompanied by a weaker side-peak at ca. 950 nm.

The most pronounced observed drop of transmittance occurs in the spectral regions outside the spectral intervals where the optical absorption bands of either the bare quinoline ligand or its Co complex reside. For interpretation of the observed spectral changes, we have to consider also optical properties of the species appearing on the right side of the reaction schema, given by Equation (1), represented here by the [Co(NH_3_)_m_]^2+^ Br_2_ complex. As already mentioned, the structure of the latter can be further modified by optional presence of other ligands. We propose that the observed decrease in transmittance is the result of the decomposition of the original reagent complex under influence of ammonia, and subsequent formation of a new complex coordinated by ammonium groups (possibly supplemented by other ligands, such as water or chlorine, whose presence in the reagent matrix cannot be excluded). The optical absorption bands corresponding to Co^2+^ d-electrons transitions in ammine complexes are known to be located at the UV-VIS spectral boundary, the position being compatible with the observed short-wavelength drop of transmittance. Increase in optical absorption at the long-wavelength side may possibly suggest that action also takes place with ligands from beginning of the spectroscopic series (such as OH^−^ or Cl^−^) characterized by a small value of ligand-field splitting parameter. Concentration change of these species during the exposition of chemo-optic transducer layer by dry ammonia gas may occur due to the strong proton absorption ability of the latter, and the known presence of water and PtCl_6_ catalyst residua in the thermally cured PDMS matrix.

## 5. Conclusions

Sensing structure based on a plasmonic EOT array fabricated by FIB technique in thin Au layer overlaid by chemo-optical transducer consisting of PDMS matrix filled with Co-DPQ organo-metallic reagent was prepared, characterized, and its spectral optical transmittance tested under NH_3_/N_2_ atmosphere of various ammonia gas concentration ranging from 0 to 1541 ppm.

Well-resolved changes of transmittance were observed conformal with the reaction of chemo-optical transducer under ammonia exposition, confirming sensing applicability of the proposed EOT-based structure. Most pronounced spectral changes appeared at the short- and long-wavelength margins of the tested spectral region 200–1200 nm, with the maxima located at ca. 350 nm, 480 nm, 950 nm, and 1100 nm. Saturation of the sensor response was observed for ammonia concentration exceeding ca. 1000 ppm.

Interpretation of the mechanism behind the observed spectral changes is proposed, based on the theoretical description of the used plasmonic nanostructure, the known chemical reaction of the reagent with ammonia gas, and the composition of the applied chemo-optical transducer.

Further experimental and theoretical research is, however, necessary in order to clarify the observed sensing mechanism in details, and test the dependence between parameters of the sensing structure and its sensitivity margins, as well as kinetics and repeatability of the detection process.

Comparing with the principles used nowadays, regarding ammonia gas sensors, the proposed EOT sensing head provides several potential assets, such as absence of electricity connections allowing for its usage in a ‘zone zero’ explosive environment, and its ease to be adapted to various local as well as non-local fiber optics interrogation schemes.

## Data Availability

Data available from the authors upon the personal request.
